# Automated detection and classification of intestinal protozoan cysts using a nuclei structure-based deep learning framework for epidemiological surveillance

**DOI:** 10.1016/j.parepi.2026.e00545

**Published:** 2026-09-11

**Authors:** Je-Chiang Tsai, Yi-Ru Chen, Shu-Min Tan, Kai-Lun Jin, Chih-Chi Wu, Jie-Hong Lai, Kuan-Lin Huang, Chia-Chieh Chu, Shih-Hsun Hung, Jiunn-Yuan Lin, Sukhdeep Singh Bal, Fan-Pei Yang, Kang-Shuo Lee, Tsai-Wei Chen, Chih-Hsing Hung, Ching-Chao Liang, Huei-Jiun Tsai, I-Pin Chen, Chiu-Hui Li, Ya-Nien Hsieh, Chin-Ling Tsai

**Affiliations:** a Department of Mathematics, National Tsing Hua University, Hsinchu, Taiwan; b Department of Applied Mathematics, National Yang Ming Chiao Tung University, Hsinchu, Taiwan; c Department of Foreign Languages and Literature, National Tsing Hua University, Hsinchu, Taiwan; d Kaohsiung Municipal Siaogang Hospital, No. 482, Shanming Rd., Siaogang Dist., Kaohsiung City 812, Taiwan; e Department of Physics, National Chung Cheng University, Chiayi, Taiwan; f Wellcome Centre for Integrative Neuroimaging, University of Oxford, Oxford, UK

**Keywords:** Intestinal protozoa, Epidemiological surveillance, Parasite control, Amebiasis, *Entamoeba* complex, Morphological screening, Explainable artificial intelligence, Neglected tropical diseases, SDG 3: Good health and well-being

## Abstract

Intestinal parasitic infections (IPIs) remain a critical global public health concern, particularly for epidemiological surveillance in resource-limited tropical regions. Standard laboratory diagnostics heavily rely on the manual microscopic examination of stool samples, a process that is notoriously time-consuming, labor-intensive, and highly dependent on the scarce availability of experienced parasitologists. To alleviate medical workloads and enhance community-wide screening efficiency, reliable, automated diagnostic tools are urgently needed. From an epidemiological control perspective, such tools should not only improve image-level classification but also support scalable screening, case triage, and prioritization of specimens requiring confirmatory testing in routine surveillance programs. However, most existing frameworks operate as end-to-end “black boxes” that rely solely on pixel-level data, significantly lacking the clinical explainability and morphological reasoning required for high-stakes medical decisions. To address this critical limitation, this study introduces an innovative hybrid framework that integrates deep learning algorithms with expert morphological domain knowledge to classify seven commonly encountered intestinal protozoan cysts (*Entamoeba histolytica* (*E. histolytica*), *Giardia lamblia*, *Entamoeba coli*, *Endolimax nana*, *Entamoeba hartmanni* (*E. hartmanni*), *Iodamoeba bütschlii*, and *Blastocystis* sp.). Utilizing a comprehensive dataset of 3143 microscopic images, the system first deploys a Mask R-CNN model to accurately detect and segment intracystic nuclei. Subsequently, a rule-based decision engine classifies the cysts by extracting key morphological attributes, including cyst shape, size boundaries, and exact nuclear configurations. The proposed framework demonstrated high diagnostic efficiency, achieving an exceptional processing speed of 0.04 s per image, making it highly scalable for high-throughput population screening. The system achieved a robust overall classification accuracy of 84.8%. While conventional light microscopy often struggles to differentiate *E. hartmanni* from *E. histolytica* due to profound structural similarities, our framework successfully resolves this taxonomic ambiguity from an epidemiological perspective. When *E. histolytica* and *E. hartmanni* were grouped into a single complex category to address this intrinsic limitation of microscopy-based methods, the overall system accuracy improved to 87.8%. This knowledge-driven AI framework provides a cost-effective, interpretable, and high-throughput screening and decision-support tool to support active disease surveillance, specimen triage, and infectious disease control programs in endemic areas.

## Graphical abstract

**Figure:**
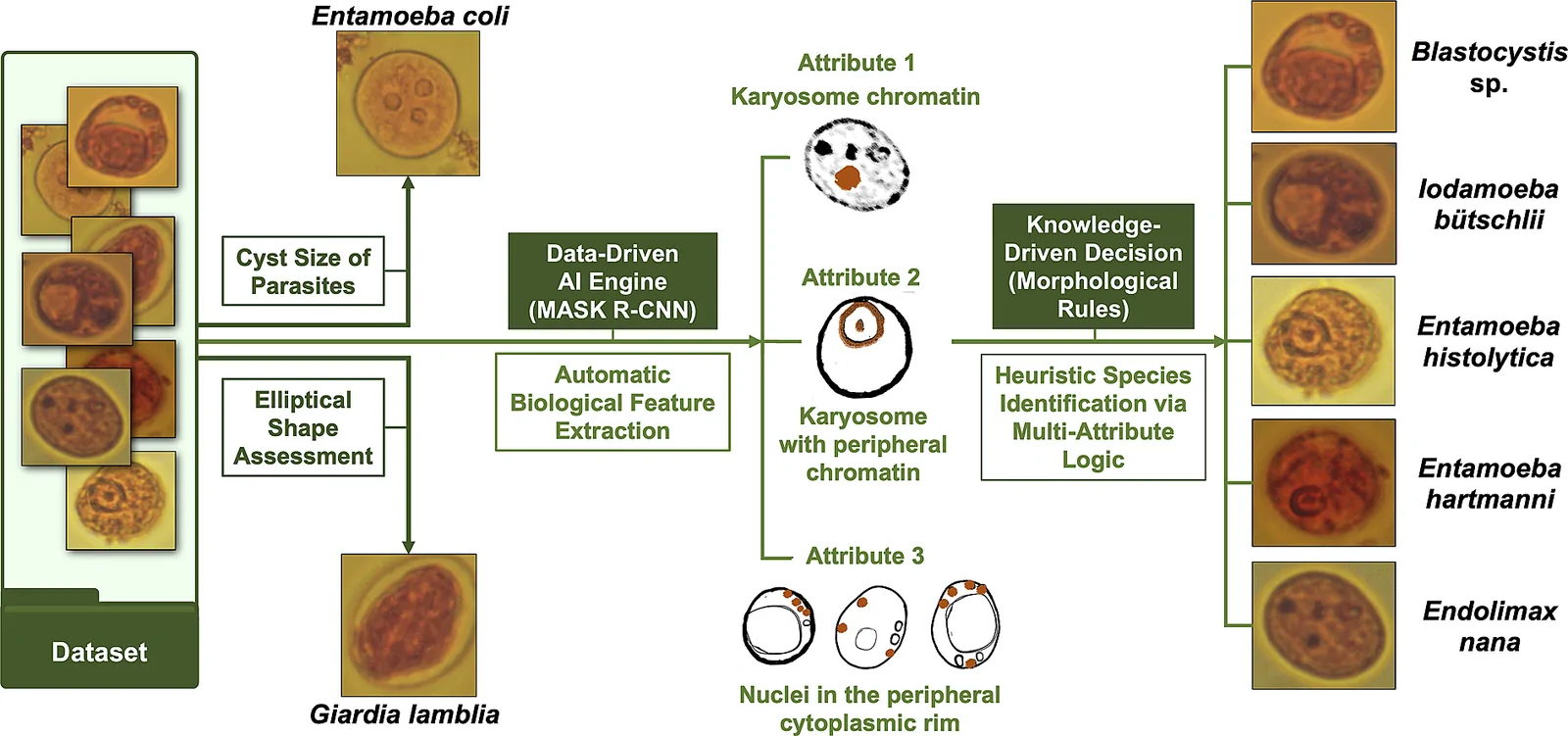

## 1 Introduction

Intestinal parasitic infections (IPIs) remain prevalent in tropical and subtropical regions, particularly in developing nations within Southeast Asia, South Asia, Africa, and Central/South America (Colley et al., 2014; World Health Organization, 2019; Ahmed, 2023; Pullan et al., 2014; Hotez et al., 2008; Olveda et al., 2013; Mortelé et al., 2004; Baert, 2007; Martínez et al., 2005; Lin and Kao, 2013; Singh et al., 2006). According to the Global Burden of Disease Study 2023, enteric infectious diseases resulted in an estimated 1.27 million deaths globally in 2023, with South Asia and sub-Saharan Africa accounting for approximately 600,000 and 500,000 deaths, respectively (Wang, 1998; GBD 2023 Diarrhoeal Disease and Enteric Infectious Diseases Collaborators, 2026). Factors such as climate, sanitation, poverty, and limited healthcare access contribute to the high frequency of IPIs in these areas (Ahmed, 2023; Kloos and Zein, 2019). Furthermore, increased globalization driven by labor migration and international travel has facilitated the increased transmission risks of IPIs (Wang, 1998; GBD 2023 Diarrhoeal Disease and Enteric Infectious Diseases Collaborators, 2026). Consequently, this shifting epidemiological landscape places unprecedented strain on national disease notification systems, demanding more robust and proactive surveillance strategies to prevent localized outbreaks in non-endemic countries.

Human intestinal parasites include helminths and protozoa, which are the causative pathogens of diseases. Helminth eggs, averaging 50 μm in size, are significantly larger than protozoan cysts, which average about 10 μm. Additionally, the structural variability and ambiguities within protozoan cysts make the manual microscopic evaluation of stool samples particularly challenging. This study focuses on amoebic protozoa, a major subgroup of protozoa. Among these, *Entamoeba histolytica* (*E.* histolytica), a key causative agent of amoebiasis, is of primary concern. Other intestinal protozoa, including amoebic species such as *Entamoeba hartmanni* (*E.* hartmanni), *Endolimax nana* (*E. nana*), *Entamoeba coli* (*E.* coli), *Iodamoeba bütschlii* (*I*. *bütschlii*), as well as *Giardia lamblia* (*G. lamblia*, a flagellated protozoan) and *Blastocystis* sp. (*Blastocystis* sp., belonging to Stramenopiles) (Stensvold and Clark, 2016; Stensvold et al., 2007) can also cause human infections (Huang et al., 2014). The primary route of IPI transmission is fecal-oral (Ahmed, 2023), involving ingestion of food or water contaminated with amoebic cysts (Mortelé et al., 2004; Baert, 2007; Martínez et al., 2005; Lin and Kao, 2013). Once ingested, these cysts develop into trophozoites that invade the mucosa of the intestinal wall (Baert, 2007; Martínez et al., 2005). The detection and diagnosis of IPIs is further complicated by the high prevalence of asymptomatic infections. These asymptomatic carriers act as hidden reservoirs within communities, silently shedding viable cysts into the environment and fueling persistent, untraced transmission chains, particularly among food handlers and institutionalized populations. Although most people infected with *E. histolytica* remain asymptomatic, some may experience symptoms such as fever, chills, abdominal pain, and bloody diarrhea. In rare cases, *E. histolytica* can invade the liver, forming abscesses, or spread to other organs, including the lungs or brain (Mortelé et al., 2004; Baert, 2007; Martínez et al., 2005; Lin and Kao, 2013; Singh et al., 2006; Taiwan Centers for Disease Control, 2017). Furthermore, *E. histolytica* is morphologically indistinguishable from the non-pathogenic *Entamoeba dispar* (*E. dispar*) by light microscopy alone (Diamond and Clark, 1993), further underscoring the need for accurate and reliable diagnostic tools.

Stool examination for intestinal parasites, such as the Merthiolate-Iodine-Formaldehyde (MIF) concentration method, is the most reliable and commonly used method for detecting various parasites (Incani et al., 2017; Sapero and Lawless, 1953). In this method, MIF-treated stool sediment is placed on an 18 × 18 mm cover slip and examined at 1000× magnification under a microscope for manual microscopy. The detection and identification of parasite cysts rely heavily on the experience and skills of medical laboratory technicians. However, there can be inter-rater variability in detection rates and accuracy among laboratory technicians, making the process subjective and time-consuming. To address these challenges, the development and implementation of an AI-powered parasitic cyst identification system offers a promising solution (Ma et al., 2023). Such systems shall not only enhance accuracy and efficiency but also provide critical support for global initiatives to control and prevent IPIs, ultimately improving public health and safeguarding human well-being worldwide.

In general, AI-based parasite identification methods utilize traditional machine learning (ML) (e.g., SVM and ANN (Jiménez et al., 2020; Nkamgang et al., 2018; Alva et al., 2017; Tchinda et al., 2018; Ray et al., 2020)) and deep learning (DL) methods, which are often built on CNN-based architectures (Suwannaphong et al., 2023; Butploy et al., 2021; Lee et al., 2022; Oliveira et al., 2022; AlDahoul et al., 2023). As per the literature, there are two main ML-based approaches for classifying protozoan cysts. The first is to use morphological characteristics (for example, size, shell, and shape) to classify protozoan cysts, which may only work if the structure of the parasite cyst is simple (Suzuki et al., 2012). The second is to use the whole image pixels, reduce the image to a smaller one, and then apply principal component analysis (PCA) to project the resulting image into a feature space of lower intrinsic dimensionality for classification (Nkamgang et al., 2018; Tchinda et al., 2018; Tchinda et al., 2015; Kumar et al., 2023a). On the other hand, state-of-the-art deep learning methods, such as CNN, YOLO models (e.g., YOLOv5 (Kumar et al., 2023b) and Tiny-YOLOv4 (Naing et al., 2022)), and the C2BNet architecture (Wan et al., 2025), can automatically extract features from parasite cysts. Moreover, DL methods have successfully classified helminth eggs. However, these DL methods may not work well for classifying protozoan cysts due to the diversity of their structures, even within a single cyst type. Moreover, most modern deep learning approaches operate as end-to-end “black boxes” that rely solely on pixel-level features. They significantly overlook the classical morphological criteria, such as precise nuclei counts and cyst size metrics, that clinical parasitologists depend on. Consequently, these pure AI models frequently misclassify morphologically cryptic species (e.g., *Entamoeba* complex) and lack the clinical explainability required for high-stakes medical diagnostics, especially in resource-limited endemic regions where expert microscopists are scarce. A recent review has highlighted that explainable AI frameworks are essential not only for improving diagnostic transparency and clinician trust, but also for ensuring that AI-driven tools remain adaptable and accessible in the low-resource settings where parasitic diseases impose the greatest burden (Tunali, 2026). This diagnostic gap heavily compromises the equity of primary healthcare delivery, as rural clinics often lack both the specialists and the infrastructure to generate reliable epidemiological data, leading to severe underreporting and delayed interventions.

To address these critical limitations and support global parasite control initiatives, this paper presents a novel, explainable two-stage hybrid framework that bridges state-of-the-art computer vision with clinical domain knowledge. The proposed system is specifically designed for the automated detection and classification of seven commonly encountered intestinal protozoan cysts (*E. histolytica*, *G. lamblia*, *E. coli*, *E. nana*, *E. hartmanni*, *I. bütschlii*, and *Blastocystis* sp.). In the first stage, a Mask R-CNN model is trained to achieve high-precision instance segmentation of intracystic nuclei. In the second stage, instead of relying on autonomous AI guessing, a rule-based expert system automatically extracts strict morphological attributes, such as cyst morphometrics and nuclei configurations, to replicate the decision-making process of senior parasitologists. Furthermore, this study explicitly addresses the inherent optical limitations of microscopy in differentiating *E. histolytica* from *E. hartmanni*, validating a reliable screening-and-triage workflow that aligns with WHO guidelines (*Wkly Epidemiol. Rec.*, 1997). Our framework achieves ultra-low computational overhead, demonstrating great potential for deployment on lightweight diagnostic devices in field-level epidemiological surveys. Therefore, the objective of this study is not merely to develop an automated image classifier, but to establish an interpretable AI-assisted screening framework that can be integrated into parasite surveillance workflows. In particular, the proposed system is designed to reduce routine microscopy workload, flag morphologically ambiguous *Entamoeba*-complex cases for confirmatory molecular testing, and support scalable monitoring of intestinal protozoan infections in public-health settings.

## 2 Methods and materials

### 2.1 Ethics statement and dataset acquisition

This study was approved by the Institutional Review Board of Kaohsiung Municipal Siaogang Hospital, Taiwan, for the anonymous use of leftover human stool specimens collected by the Laboratory of Medicine of Kaohsiung Municipal Siaogang Hospital, Taiwan (Approved No. KMUHIRB-E(II)- 20,240,334). All the procedures were strictly followed the hospital's guidelines and regulations.

The database comprised 3143 images representing seven distinct species. All fecal smear specimens were prepared and processed using standard clinical parasitological protocols and stained with Lugol's iodine (as part of the MIF protocol) to clearly delineate the internal nuclei structures of the cysts. Microscopic images were captured under a standard light microscope at 1000× magnification. Specifically, stool sediment samples were examined using a wet-mounted microscope equipped with a 10× eyepiece and a 100× oil-immersion objective lens to achieve 1000× magnification on an 18 × 18 mm cover slip. The images, collected during health examinations of employed foreigners at the Laboratory of Medicine, Kaohsiung Municipal Siaogang Hospital, Taiwan, were labeled by medical laboratory experts.

The inclusion of specimens obtained from routine health examinations of employed foreign workers reflects an important real-world surveillance context in Taiwan, where stool examinations are used as part of public health monitoring for intestinal parasitic infections. Because population mobility can influence the epidemiology of IPIs, improving diagnostic workflows in such routine screening programs may strengthen early detection and public health responses.

### 2.2 Ground truth verification and laboratory criteria

Ground truth labels for all parasite cyst images were established through a two-stage process. In the first stage, stool sediment specimens were examined by experienced medical laboratory technicians at the Laboratory of Medicine, Kaohsiung Municipal Siaogang Hospital, Taiwan, using the Merthiolate-Iodine-Formaldehyde (MIF) concentration method at 1000× magnification. Cyst identification was based on established microscopic morphology criteria (Peters and Pasvol, 2002; Jeon, 1973).

In the second stage, all cases involving *E. histolytica* were subjected to mandatory confirmatory molecular diagnosis through Taiwan's statutory notifiable disease reporting system. Suspected cases were reported to the Taiwan Centers for Disease Control (Taiwan CDC), which performed confirmatory polymerase chain reaction (PCR) analysis targeting the small subunit ribosomal DNA (SSU rDNA / 18S rDNA) locus (Tanyuksel and Petri, 2003; Fotedar et al., 2007). This molecular approach is the established gold standard for differentiating *E. histolytica* from morphologically identical but nonpathogenic species, including *E. dispar*, *Entamoeba moshkovskii* (*E. moshkovskii*), and *Entamoeba polecki* (*E. polecki*) (Diamond and Clark, 1993; *Wkly Epidemiol. Rec.*, 1997). Only cases assigned official “confirmed case” status by Taiwan CDC under the statutory notifiable disease reporting system (Law on the Prevention and Control of Infectious Diseases, Taiwan) were included in the dataset as *E. histolytica* ground truth labels (Taiwan Centers for Disease Control, 2017). This design links model validation directly to an operational surveillance system rather than to image annotation alone, thereby increasing the public-health relevance of the proposed framework. By embedding the dataset ground truth directly within Taiwan's official infectious disease containment network, the labels for *E. histolytica* carry substantial regulatory weight. This alignment with government-mandated epidemiologic surveillance protocols ensures that the validation of our computational model is directly benchmarked against national public health standards.

It is acknowledged that morphological examination alone cannot differentiate *E. histolytica* from *E. dispar* or related species (Diamond and Clark, 1993). Therefore, PCR-confirmed diagnoses from the Taiwan CDC provide a robust, legally validated ground truth for the *E. histolytica* class in this study. For the remaining six parasite classes, morphological identification by trained laboratory personnel, following standardized diagnostic criteria, served as the ground truth (Peters and Pasvol, 2002).

**Fig. 1:**
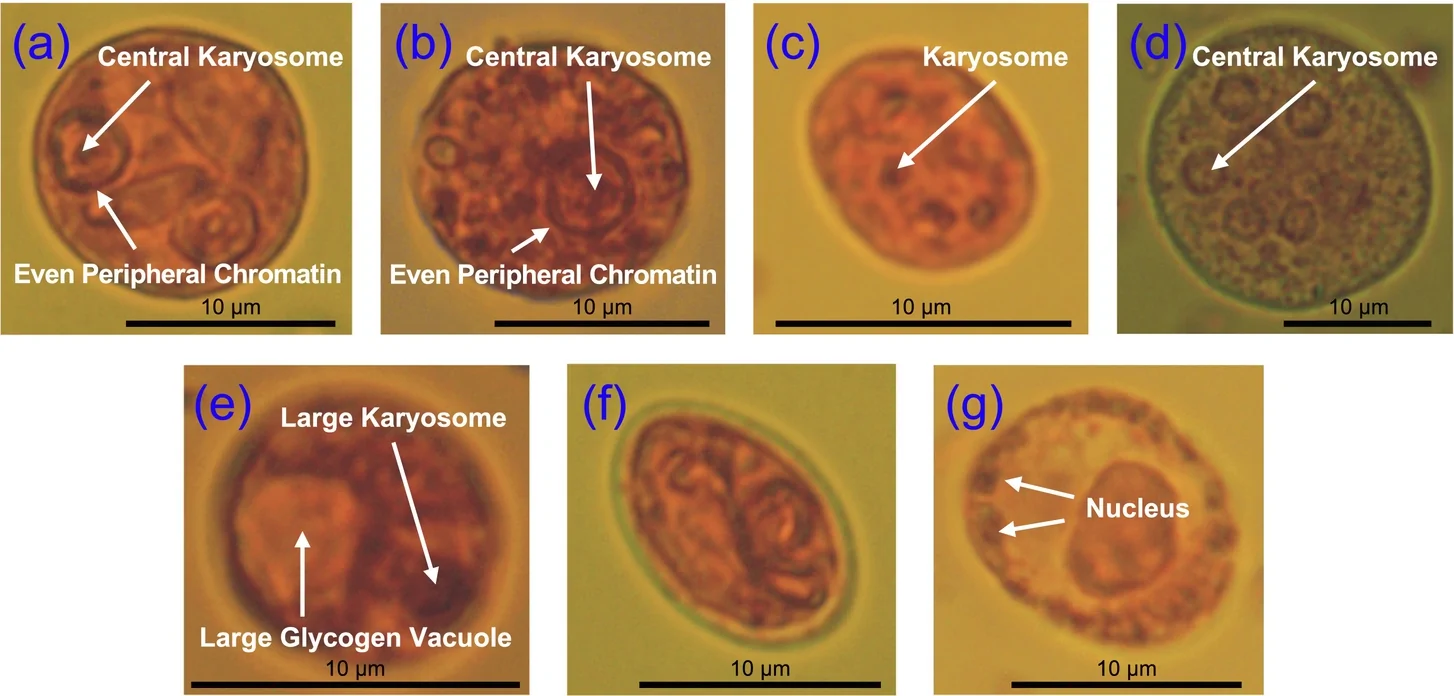
Representative microscopic images of seven intestinal protozoan types identified in this study. Scale bars = 10 μm. (a). *Entamoeba histolytica*, showing central karyosome, and even peripheral chromatin; (b). *Entamoeba hartmanni*, showing central karyosome with peripheral chromatin; (c). *Endolimax nana*, showing distinct karyosomes without peripheral chromatin; (d). *Entamoeba coli*, showing central karyosomes and multiple nuclei; (e). *Iodamoeba bütschlii*, showing a large karyosome and a prominent glycogen vacuole; (f). *Giardia lamblia*, identified by its characteristic oval shape (elongated axis); no intracellular structures are annotated as classification relies solely on morphometric parameters (axis ratio > 1.36); (g). *Blastocystis* sp. (vacuolar form), showing central body and peripheral nuclei.

Usually, seven classes of intestinal parasite cysts are identified in human stool samples during health examinations of employed foreigners in Taiwan (Fig. 1). The microscopic morphology characteristics of these seven parasite cysts are as follows (Peters and Pasvol, 2002; Jeon, 1973):

1. *Entamoeba histolytica* cyst: The cyst is spherical, typically measuring 12–15 μm in diameter, and usually contains one to four nuclei. Each nucleus has a small, centrally located karyosome with finely and evenly distributed peripheral chromatin.
2. *Entamoeba hartmanni* cyst: The cyst is spherical, measuring 6–8 μm in diameter, and typically contains one to four nuclei. Slightly smaller than the *E. histolytica* cyst, each nucleus has a small, centrally positioned karyosome with finely and evenly distributed peripheral chromatin.
3. *Endolimax nana* cyst: The cyst is round or oblong, typically measuring 6–8 μm in diameter, and usually contains four nuclei with distinct karyosome chromatin and no peripheral chromatin.
4. *Entamoeba coli* cyst: The cyst is round and typically measures 15–25 μm in diameter, making it the largest among the seven types. It generally contains eight nuclei with prominent karyosome chromatin.
5. *Iodamoeba bütschlii* cyst: The cyst is round or oval, typically measuring 10–12 μm in diameter, with a large, usually central karyosome chromatin and no peripheral chromatin. A prominent glycogen vacuole occupies most of the cyst, displacing the nucleus to one side.
6. *Giardia lamblia* cyst: The cyst is oval-shaped with an elongated axis, featuring a dark and uniformly colored interior.
7. *Blastocystis* sp. cyst: The cyst contains a large vacuole with numerous transparent regions, pushing its nuclei to the periphery of the cyst. *Blastocystis* sp. exists in multiple morphological forms, including vacuolar, granular, amoeboid, and cyst forms (Stensvold and Clark, 2016; Tan, 2008). The present study focuses on the **vacuolar form**, which is the most frequently encountered form in MIF-stained clinical stool specimens and is characterized by a large vacuole and nuclei compressed to the periphery of the cyst (formally defined as Attribute 3 in Section 2.4.3, Knowledge-based heuristic cross-check rules). The performance of the proposed algorithm on other morphological forms of *Blastocystis* sp. has not been evaluated and is identified as a direction for future work.

### 2.3 Image preprocessing and dataset splitting

#### 2.3.1 Data preprocessing

As the images were in 8-bit format, normalization was performed by converting the pixel values to floats and dividing them by 255.

#### 2.3.2 Image segmentation

The procedure efficiently identifies parasite cysts in medical images through preprocessing, OTSU binarization, and erosion operations. By detecting contours and applying shape-based filtering, it accurately selects parasite cysts.

#### 2.3.3 Labels and dataset splitting

The image dataset includes seven label types, as shown in Table 1 below. It is randomly split into two subsets: the training set, comprising 80% of the data for model training, and the test set, comprising the remaining 20% for model evaluation.

**Table 1:** Seven classes of intestinal parasite cysts and their corresponding image counts.

Parasite cyst	Training set	Test set	Total number of images
*E. histolytica*	107	85	192
*E. hartmanni*	71	51	122
*E. nana*	322	119	441
*E. coli*	50	47	97
*I. bütschlii*	13	11	24
*G. lamblia*	126	118	244
*Blastocystis* sp.	1718	305	2023
Total	2407	736	3143

### 2.4 Two-stage hybrid diagnostic framework

**Fig. 2:**
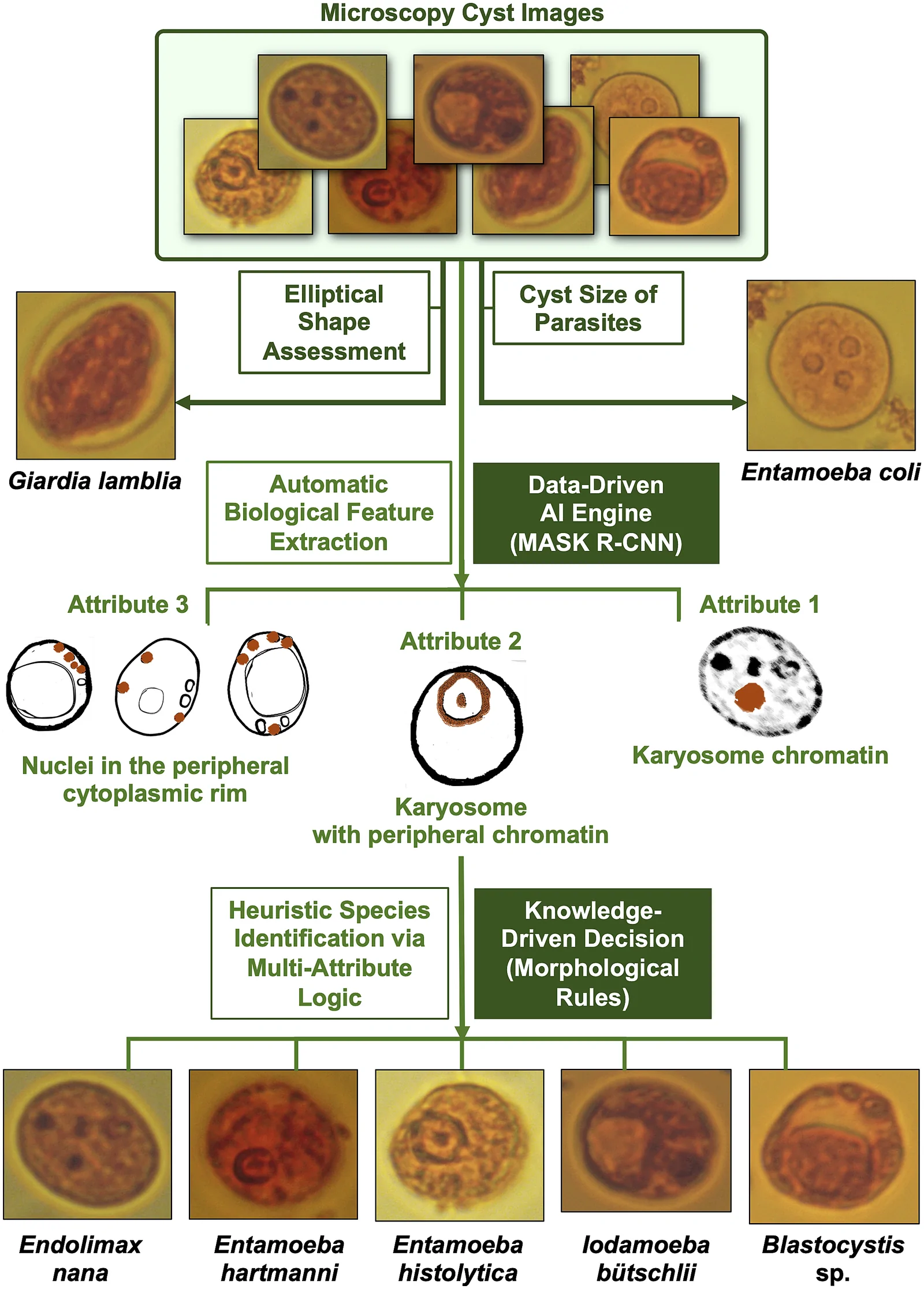
The proposed explainable two-stage hybrid framework for automated protozoan cyst classification.

The proposed hybrid diagnostic pipeline for classifying intestinal protozoan cysts is structured as a dual-stage decision framework (Fig. 2). Upon receiving a candidate cyst image as input, Stage 1 deploys a preliminary morphological filtration mechanism that screens size and shape descriptors to robustly isolate and classify *E. coli* and *G. lamblia* cysts. For the remaining unclassified specimens, Stage 2 employs a fine-tuned Mask R-CNN to detect and precisely localize three defined intracellular nuclei attributes. These extracted structural attributes are subsequently routed through a knowledge-based, cross-check heuristic classifier to execute the final species prediction. The detailed technical execution of each stage is delineated in the following subsections. This two-stage approach was strategically developed to balance clinical accuracy with computational efficiency, making it highly suitable for point-of-care integration. By offloading computational strain during the preliminary filtration stage, the framework demonstrates a viable pathway for deployment on lightweight, field-portable hardware, which is essential for conducting active, wide-scale epidemiological screenings in remote or resource-limited endemic zones.

The cyst identification scheme consists of three steps, detailed as follows:

#### 2.4.1 Step 1: preliminary morphological filtration

This step utilizes basic morphological features to identify *E. coli* and *G. lamblia* cysts. Two key parameters are defined:

*Parameter 1 (size parameter):* Measures the parasite cyst diameter (used when the cyst is approximately circular).

*Parameter 2 (oval parameter):* Represents the ratio of the primary axis length to the minor axis length (used when the cyst is approximately elliptical).

These parameters form the basis of the following filtration criteria:

1. Filtering *E. coli* cysts:
  • *E. coli* cysts, characterized by their large and circular shape, are identified using the size parameter (Parameter 1).
  • If Parameter 1 exceeds 15 μm, the cyst is classified as an *E. coli* cyst. Otherwise, it is not.
2. Filtering *G. lamblia* cysts:
  • *G. lamblia* cysts, known for their oval shape, are identified using the oval parameter (Parameter 2).
  • If Parameter 2 exceeds 1.36, the cyst is classified as a *G. lamblia* cyst. Otherwise, it is not.

According to (Swiss TPH, 2026; Lehman, 2008; HOSPITAL C-YC, 2026), the cysts of *E. coli* typically measure 15 to 25 μm in diameter, although sizes ranging from 10 to 30 μm have also been reported. A threshold of 15 μm was adopted in this algorithm as it represents the lower bound of the commonly reported size range, providing a practical balance between sensitivity and specificity in distinguishing *E. coli* cysts from smaller intestinal protozoa. This threshold is further supported by the fact that the other protozoan species in this study (*E. histolytica* 12–15 μm, *E. hartmanni* 6–8 μm, *E. nana* 6–8 μm, and *I. bütschlii* 10–12 μm) all fall below or at the lower bound of this cutoff, confirming that 15 μm provides a biologically meaningful separation between *E. coli* and the remaining species (Peters and Pasvol, 2002; Jeon, 1973). Together, these criteria provide a preliminary screening process for *E. coli* and *G. lamblia* cysts. Additionally, the size parameter (Parameter 1) can further aid in distinguishing among *E. histolytica*, *E. hartmanni*, and *E. nana* cysts, thereby reducing the computational cost for subsequent AI-based predictions (as described in **Step 3**).

#### 2.4.2 Step 2: locating intracellular features via Mask R-CNN

Basic morphological features alone cannot distinguish all types of cysts. For example, *E. hartmanni* and *E. nana* cysts share a similar size range and typically exhibit round shapes. To differentiate these and other cyst types, we rely on three specific intracellular morphological attributes.

**Attribute 1:** The nucleus possesses a karyosome chromatin and no peripheral chromatin.

**Attribute 2**: The nucleus has a small, generally round and central or eccentric karyosome with fine, evenly distributed peripheral chromatin.

**Attribute 3:** The nuclei are compressed to the periphery of the cyst.

The attributes associated with each type of cyst are summarized in Table 2 below.

**Table 2:** Comparison of Attributes 1, 2, and 3 Among Different Cyst Types.

	*E. histolytica*	*E. hartmanni*	*E. nana*	*I. bütschlii*	*Blastocystis* sp.
Attribute 1			✓	✓	
Attribute 2	✓	✓			
Attribute 3				✓	✓

**Fig. 3:**
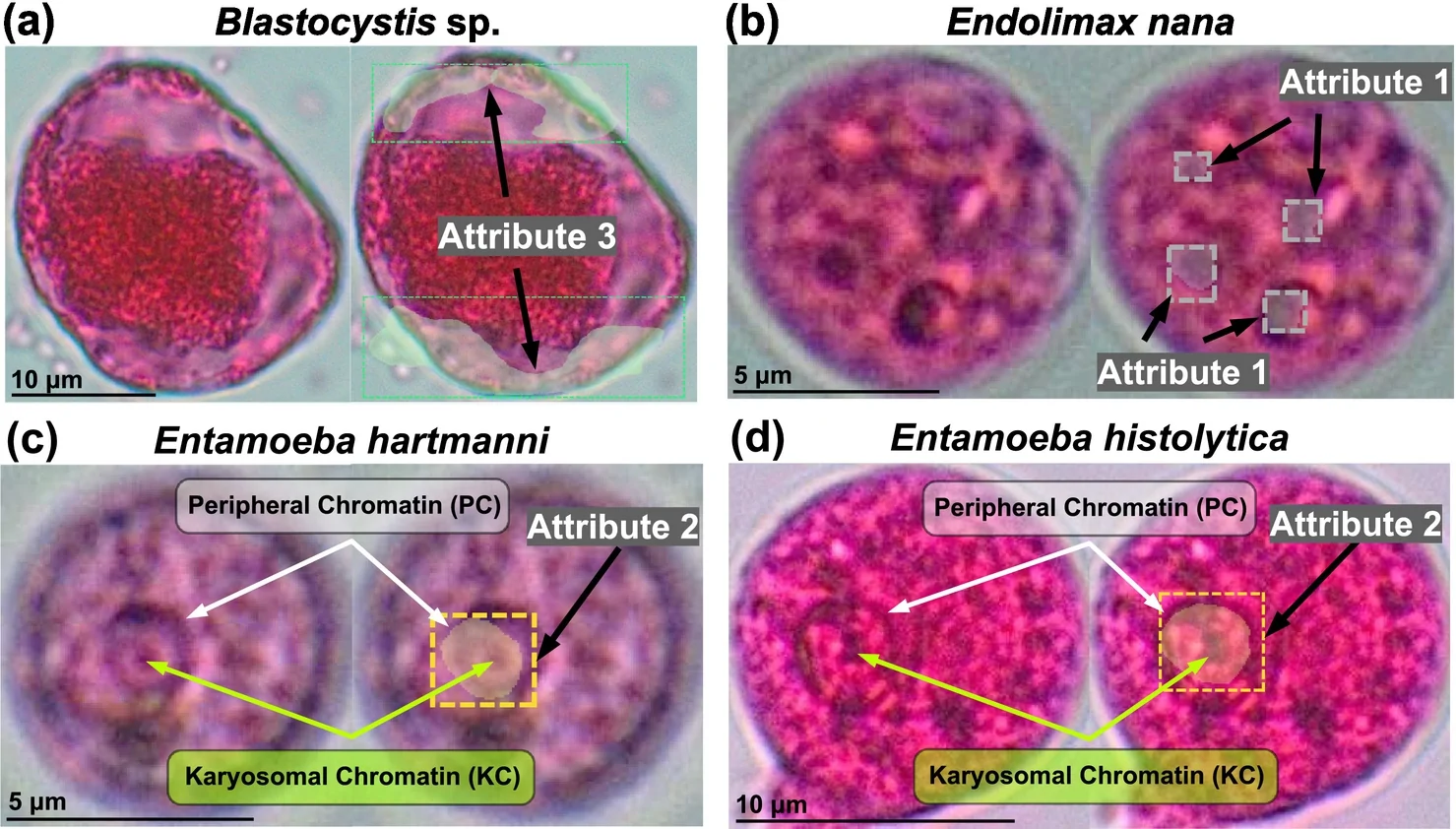
Microscopic images of various cysts with detected attribute features. Each panel pair (left and right) represents the same cyst. Left panels: Original microscopic images of the cysts. Right panels: Regions identified by Mask R-CNN detection. Details are as follows: (a). Detection of Attribute 3 in *Blastocystis* sp. cyst. (b). Detection of Attribute 1 in *Endolimax nana* cyst. (c). Detection of Attribute 2 in *Entamoeba hartmanni* cyst, highlighting Peripheral Chromatin (PC) and Karyosomal Chromatin (KC). (d). Detection of Attribute 2 in *Entamoeba histolytica* cyst, emphasizing Peripheral Chromatin (PC) and Karyosomal Chromatin (KC).

For each cyst image, a Mask R-CNN architecture was implemented using Detectron2 (Wu et al., 2019), a state-of-the-art object detection and segmentation library developed by Facebook AI Research. The Mask R-CNN is trained to detect three nuclear attributes. Mask R-CNN, short for Mask Region-based Convolutional Neural Network (He et al., 2017), enables precise attribute detection. As shown in Fig. 3, Detectron2 effectively locates the attributes within the cyst images. If a cyst image contains only Attribute 1, it is classified as an *E. nana* cyst. However, if Attributes 2 or 3 are present, further analysis is required, as outlined in **Step 3**.

#### 2.4.3 Step 3: knowledge-based heuristic cross-check rules

**Fig. 4:**
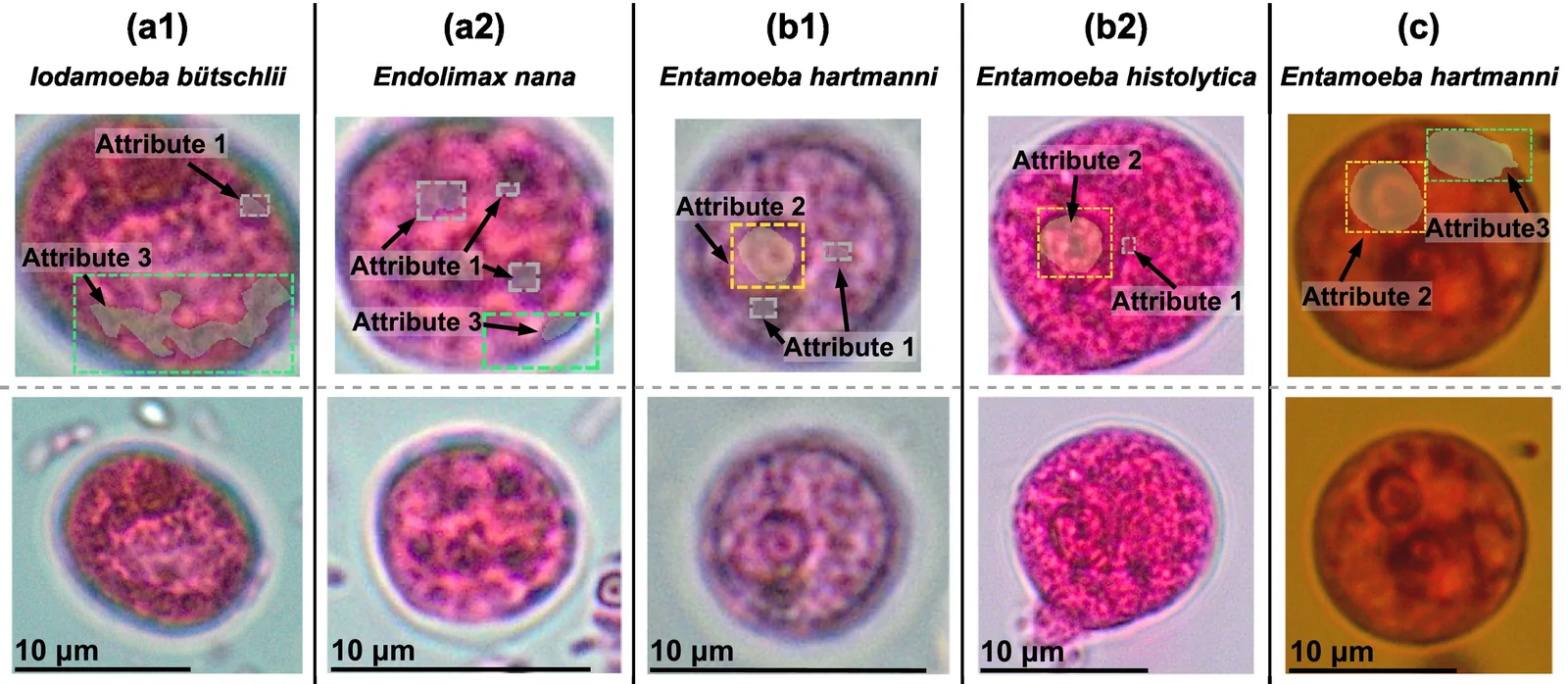
Microscopic images of various cysts with detected attribute features illustrating two-attribute cases. Each panel pair shows the original microscopic image (bottom) and the corresponding Mask R-CNN detection result (top). (a). Detection of Attributes 1 and 3 in *Iodamoeba bütschlii* (a1) and *Endolimax nana* (a2); classification is resolved by nuclei count (Case 2c, Step 3). (b). Detection of Attributes 1 and 2 in *Entamoeba hartmanni* (b1) and *Entamoeba histolytica* (b2); Attribute 1 in both panels represents a noise-induced false detection, as Attributes 1 and 2 cannot biologically coexist within a single cyst (see Table 2). In accordance with Case 2a (Step 3), Attribute 2 is prioritized over Attribute 1 due to its greater robustness to image noise, and final classification is determined by cyst size (Parameter 1 > 10μm). (c). Detection of Attributes 2 and 3 in *Entamoeba hartmanni*; similarly, Attribute 3 represents a noise-induced false detection, and Attribute 2 is prioritized for classification (Case 2b, Step 3).

Table 2 defines the biologically expected attribute profile for each cyst type under ideal imaging conditions. For example, *E. hartmanni* is expected to exhibit only Attribute 2, and *E. nana* only Attribute 1. However, in practice, Mask R-CNN may simultaneously detect two attributes within a single cyst image due to image noise, as illustrated in Fig. 4. It is important to note that such two-attribute detections do not reflect true biological coexistence: upon examining all sample cysts, we confirm that Attributes 1, 2, and 3 never biologically coexist within a single cyst. Rather, any apparent coexistence is attributable to noise-induced false detections, highlighting the limitations of relying solely on Mask R-CNN for accurate classification. To address this, we supplement the Mask R-CNN output with a cross-check procedure that incorporates additional morphological parameters, including cyst size (Parameter 1) and nuclei count, to resolve ambiguous two-attribute cases and arrive at a definitive classification. The analysis is categorized into two main cases: (1) only a single attribute is present in the cyst, and (2) two attributes are simultaneously detected due to image noise.

Case 1. Only a single attribute is present. See Fig. 3 for an illustration.
  1a. **Only Attribute 1 is present.**

According to Table 2, the corresponding parasite is *E. nana*. See Fig. 3 (b) for an illustration.

1b. **Only Attribute 2 is present.**

The corresponding parasite is either *E. histolytica* or *E. hartmanni*, according to Table 2. Next, the *E. histolytica* cyst (Class 1), which is larger than the other classes, can be identified by checking whether the associated Parameter 1 is greater than 10 μm. See Fig. 3 (c) and (d) for an illustration.

1c. **Only Attribute 3 is present.**

According to Table 2, the corresponding parasite is *Blastocystis* sp. See Fig. 3 (a) for an illustration.

Case 2. **Presence of Two Attributes.**

In this scenario, Attribute 2, a defining characteristic of *E. histolytica* cyst images, is prioritized. This decision aligns with the World Health Organization (WHO) classification of *E. histolytica* as a high-priority infectious disease pathogen (*Wkly Epidemiol. Rec.*, 1997). The parasite causes amebiasis, encompassing intestinal infections like amebic dysentery and invasive diseases such as liver abscesses (World Health Organization, 2023). To proceed, this scenario will be divided into the following three subcases:

2a. **Only Attributes 1 and 2 are present.**

According to Table 2, Attributes 1 and 2 cannot coexist within the image of a single cyst, and any apparent coexistence is likely due to image noise. The possible parasites in this case are *E. histolytica*, *E. hartmanni*, or *E. nana*. Since Attribute 2 is more robust to noise than Attribute 1 and is prioritized, the classification narrows to *E. histolytica* or *E. hartmanni*. To distinguish between the two, the cyst size is evaluated: If Parameter 1 exceeds 10 μm, the parasite is identified as *E. histolytica*. Otherwise, it is classified as *E. hartmanni*. See Fig. 4 (b1) and (b2) for an illustration.

2b. **Only Attributes 2 and 3 are present.**

As in Case 2a, the coexistence of these two attributes is primarily due to noise in the underlying images. Since Attribute 2 remains robust against noise in the cyst images, the parasite is either *E. histolytica* or *E. hartmanni,* as indicated in Table 2. As before, their inherent size difference serves as a reliable criterion for effective discrimination. See Fig. 4 (c) for an illustration.

2c. **Only Attributes 3 and 1 are present.**

**Fig. 5:**
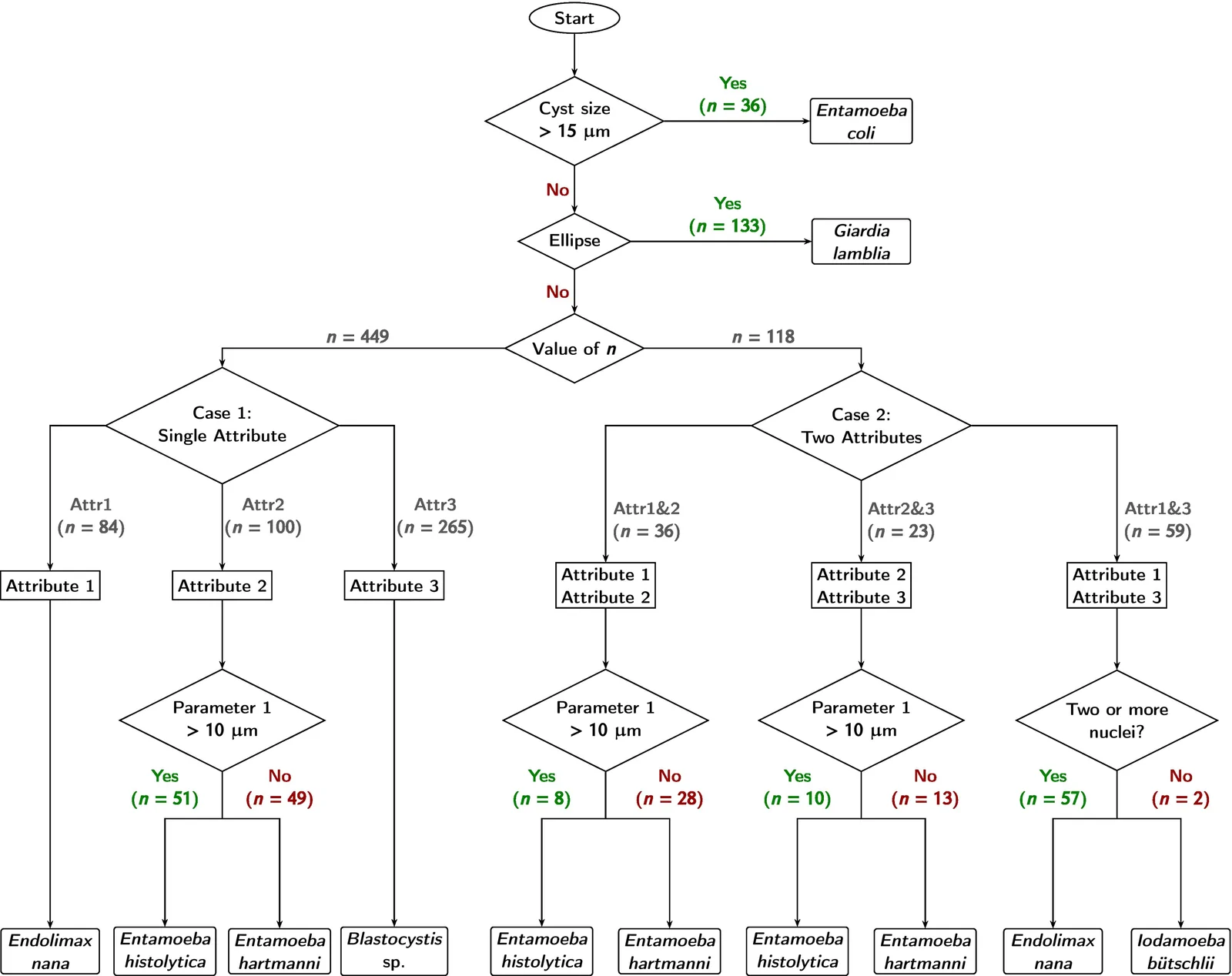
Algorithm flowchart. The flowchart visually depicts the decision-making process based on cyst attributes. It categorizes cases into single-attribute and two-attribute cases while guiding the identification of corresponding parasites. The value *n* denotes the number of test set images processed at each decision node (total: *n* = 736). Classification of *Blastocystis* sp. is limited to Case 1c; see Discussion for details.

Based on Table 2, the parasite is either *E. nana* or *I. bütschlii*. To accurately identify the parasite, additional information about the nuclei is required. *E. nana* typically has four nuclei with Attribute 1. Therefore, if two or more nuclei with Attribute 1 are detected, the parasite is identified as *E. nana*. Otherwise, it is classified as *I. bütschlii*. See Fig. 4 (a1) and (a2) for an illustration. The overall algorithm flowchart is given in Fig. 5.

### 2.5 Evaluation metrics

Evaluation metrics are essential for optimizing classifiers in deep learning tasks involving detection and classification. Commonly used metrics include Precision, Recall, and F1-score. These metrics rely on key concepts such as True Positive (TP), which refers to instances where the model correctly predicts positive values; False Positive (FP), where the model incorrectly predicts a negative instance as positive; False Negative (FN), representing cases where the model fails to predict a positive instance and classifies it as negative; and True Negative (TN), where negative values are correctly predicted. The following are the formulas for class-specific and overall metrics (Sokolova and Lapalme, 2009):

• Class-Specific Precision ($=\frac{\mathrm{TP}}{\mathrm{TP}+\mathrm{FP}}$) focuses on the proportion of correct predictions for a specific class among all instances predicted of that class.
• Class-Specific Recall ($=\frac{\mathrm{TP}}{\mathrm{TP}+\mathrm{FN}}$) measures the proportion of actual instances of a class correctly identified by the model.
• Class-Specific F1-Score ($=2\times \frac{\text{Precision}\times \text{Recall}}{\text{Precision}+\text{Recall}}$) provides a harmonic mean of Precision and Recall for a specific class.
• Overall Accuracy $\left(=\frac{\mathrm{Sum}\;\text{of}\;\mathrm{All}\;\text{True Positive}\;\left(\mathrm{TP}\right)}{\text{Total Instances in the Dataset}}\right)$ represents the proportion of correctly classified instances across all classes.
• Overall F1-Score is computed as the micro-averaged F1-Score across all classes using averaging techniques:
  $$\text{Overall}\;F1‐\text{Score}=\frac{\sum_{i=1}^{N}{\mathrm{TP}}_{i}}{\sum_{i=1}^{N}{\mathrm{TP}}_{i}+{\mathrm{FP}}_{i}+{\mathrm{FN}}_{i}}$$

In the formulas above, the subindex i denotes a specific testing class, while N represents the total number of classes analyzed (7 in this paper).

## 3 Results

The proposed algorithm achieved an overall accuracy of 84.8% and an F1-score of 0.735 on the test set, comprising 736 images. The total processing time for these images was approximately 30 s, equating to approximately 0.04 s per image, highlighting the framework's high-throughput capability. Detailed performance metrics for each protozoan class are summarized in Table 3.

**Table 3:** Class-wise accuracy, precision, recall, and F1-score.

Protozoa class	Precision (%)	Recall (%)	F1-score
*E. histolytica*	85.5	69.4	0.766
*E. hartmanni*	54.4	96.1	0.696
*E. nana*	82.3	97.5	0.889
*E. coli*	83.3	63.8	0.723
*I. bütschlii*	100.0	18.2	0.308
*G. lamblia*	81.2	91.5	0.860
*Blastocystis* sp.	98.1	85.2	0.912

The confusion matrix (Table 4) highlights the distribution of predictions across all classes.

**Table 4:** Confusion matrix.

	Actual Class							
		*E. histolytica*	*E. hartmanni*	*E. nana*	*E. coli*	*I. bütschlii*	*G. lamblia*	*Blastocystis* sp.
Prediction class	*E. histolytica*	59	2	0	7	0	0	1
	*E. hartmanni*	20	49	1	10	0	4	6
	*E. nana*	0	0	116	0	3	4	18
	*E. coli*	6	0	0	30	0	0	0
	*I. bütschlii*	0	0	0	0	2	0	0
	*G. lamblia*	0	0	2	0	3	108	20
	*Blastocystis* sp.	0	0	0	0	3	2	260

The class-wise performance metrics, including accuracy, precision, and recall for each of the seven parasite species, are detailed in Table 3. The overall F1-score of 0.735 reflects the inherent diagnostic challenges associated with certain parasite classes. Specifically, *E. hartmanni* exhibited low precision (54.4%), as its small cyst size and nuclear configuration overlap considerably with those of *E. histolytica*, leading to frequent misclassification between the two species. *I. bütschlii* had a low recall of 18.2% due to its small test set (*n* = 11) and class imbalance. However, the corresponding precision for *I. bütschlii* was a flawless 100%, demonstrating that the knowledge-based heuristic rules act as a highly conservative filter. In disease control scenarios, this stringent gating prevents false-positive alarms for rare, non-pathogenic species, thereby minimizing unnecessary psychological burden on patients and safeguarding diagnostic resources. *Blastocystis* sp. also showed a relatively high false-negative rate, attributable to noise-induced misrouting into Two-Attribute cases that do not produce a *Blastocystis* sp. output (see Discussion). Despite these challenges, this study maintains separate classifications for *E. histolytica*, *E. hartmanni*, and *E. coli*, as grouping them would obscure clinically important distinctions, particularly given the severe health complications associated with *E. histolytica* infection.

To further evaluate how morphology-based ambiguity affects surveillance performance, a surveillance-oriented grouping analysis was conducted by merging *E. histolytica* and *E. hartmanni* into a single *Entamoeba*-complex category. Consequently, the overall classification accuracy improved to 87.8%, with an overall F1-score of 0.807 (Table 5). Notably, the merged *E. histolytica*/*E. hartmanni* class achieved a recall of 95.6% and an F1-score of 0.882. This significant improvement highlights that the primary source of misclassification lies in the morphological similarity between these two species, which is an inherent limitation of microscopy-based diagnosis rather than a shortcoming of the proposed algorithm. This substantial performance gain validation supports a practical two-tier screening protocol for public health programs. By grouping these morphologically identical species during primary AI microscopy screening, health authorities can deploy a highly reliable, low-cost filter (87.8% accuracy) to capture the *Entamoeba* complex on a large scale, before escalating only the flagged cases to resource-intensive molecular confirmation. Medical practitioners often recommend PCR testing when these two species are encountered, as *E. histolytica* is a dangerous pathogen that can cause severe health complications, according to WHO guidelines (*Wkly Epidemiol. Rec.*, 1997). Incorporating this verification step ensures accurate identification and management of *E. histolytica* infections.

**Table 5:** Class-wise accuracy, precision, recall, and F1-score with *Entamoeba histolytica* & *Entamoeba hartmanni*, treated as a single species.

Protozoa class	Precision (%)	Recall (%)	F1-score
*E. histolytica* & *E. hartmanni*	81.8	95.6	0.881
*E. nana*	82.3	97.5	0.893
*E. coli*	83.3	63.8	0.723
*I. bütschlii*	100.0	18.2	0.308
*G. lamblia*	81.2	91.5	0.860
*Blastocystis* sp.	98.1	85.2	0.912

The adjusted confusion matrix (Table 6) reflects these combined categories and their respective performance.

**Table 6:** Confusion matrix with *Entamoeba histolytica* & *Entamoeba hartmanni*, treated as a single species.

	Actual Class						
		*E. histolytica* & *E. hartmanni*	*E. nana*	*E. coli*	*I. bütschlii*	*G. lamblia*	*Blastocystis* sp.
Prediction Class	*E. histolytica* & *E. hartmanni*	130	1	17	0	4	7
	*E. nana*	0	116	0	3	4	18
	*E. coli*	6	0	30	0	0	0
	*I. bütschlii*	0	0	0	2	0	0
	*G. lamblia*	0	2	0	3	108	20
	*Blastocystis* sp.	0	0	0	3	2	260

## 4 Discussion

Intestinal parasitic infections (IPIs) are prevalent in tropical and subtropical regions, posing diagnostic challenges due to manual, time-consuming, and inconsistent microscopic analyses with low accuracy. Intestinal protozoa, particularly amoebic species, with their small cyst size and structural variability, further complicate diagnosis. The proposed method demonstrates significant potential for automating intestinal parasite diagnosis, achieving high accuracy and efficiency. By integrating Mask R-CNN for nuclei structure detection and a cross-check classification approach, the model successfully addresses many challenges posed by the structural diversity of protozoan cysts. From a public health perspective, the reliance on manual microscopy not only creates clinical bottlenecks but also severely hinders large-scale epidemiological mapping and active community surveillance, particularly in resource-limited endemic regions. By providing an objective, high-throughput tool, the proposed framework offers a scalable solution to accelerate field screening and improve the timeliness of parasitic disease reporting.

AI image recognition and classification models have traditionally employed Convolutional Neural Networks (CNNs). In the initial stages of development, we adopted the VGG16 CNN architecture, a well-established model, for classification. However, the results proved suboptimal, with an accuracy rate falling below 70%. We suspect this was due to significant variability and ambiguity in the intracellular structure of protozoan cysts, even within the same protozoan type. For instance, one of the major attributes of *Blastocystis* sp. cysts is the presence of vacuoles, yet the shape of these vacuoles varies greatly among individual cysts. Consequently, achieving high accuracy with CNN-based architectures would require an extensive dataset of cyst images. Our results demonstrate that the proposed method is promising for fully automated enteroparasitosis diagnosis and effectively addresses these challenges.

One important consideration is the clinical approach when encountering *E. histolytica* and *E. hartmanni*. Given the significant risk posed by *E. histolytica* as a dangerous pathogen (as highlighted by WHO guidelines (*Wkly Epidemiol. Rec.*, 1997)), medical practitioners often recommend further Polymerase Chain Reaction (PCR) testing to confirm diagnoses. This practice ensures that *E. histolytica* infections, which can cause severe health complications, are accurately identified and managed.

Operationally, the proposed framework may be most appropriately used as a first-line screening and triage tool within parasite control programs. In this workflow, images classified as *Entamoeba*-complex or otherwise epidemiologically relevant categories can be prioritized for expert review or confirmatory PCR, whereas high-confidence low-risk detections can be processed with reduced manual workload. Such a tiered strategy may be particularly useful for routine stool screening programs, migrant-worker health examinations, field surveys, and resource-limited settings where trained parasitologists and molecular diagnostic capacity are not readily available. From an epidemiological standpoint, such computational efficiency is highly valuable for processing large volumes of specimens during active field surveillance and outbreak investigations. In routine screening programs, this throughput may enable the system to serve as a first-line digital prescreening tool, allowing expert microscopists to focus on ambiguous or high-risk cases rather than manually reviewing all images.

A fundamental limitation of the present study, and of morphology-based diagnostic systems in general, is the inherent inability to differentiate *E. histolytica* from morphologically indistinguishable but non-pathogenic species, namely *E. dispar*, *E. moshkovskii*, and *E. polecki*, on the basis of microscopic examination alone (Diamond and Clark, 1993; Tanyuksel and Petri, 2003). These species share identical cyst morphology, making definitive identification impossible without molecular methods (Fotedar et al., 2007). This distinction is clinically critical, as *E. histolytica* is a pathogenic species responsible for amoebiasis and potentially fatal complications, including liver abscess and cerebral involvement, while *E. dispar* is considered non-pathogenic in immunocompetent individuals (*Wkly Epidemiol. Rec.*, 1997).

To address this limitation in the current study, all *E. histolytica* cases were confirmed through Taiwan's statutory notifiable disease reporting system, with PCR targeting the SSU rDNA / 18S rDNA locus performed by the Taiwan Centers for Disease Control (Taiwan CDC) as the gold standard for species confirmation (see Section 2.2, Ground truth verification and laboratory criteria). Nevertheless, in settings where PCR confirmation is unavailable, the proposed algorithm should be interpreted as a morphological screening tool rather than a definitive diagnostic system. Clinical adoption of this algorithm should therefore be accompanied by a clear workflow that flags *E. histolytica*-suspected cases for mandatory PCR confirmation, consistent with WHO guidelines (*Wkly Epidemiol. Rec.*, 1997) and current clinical practice (Tanyuksel and Petri, 2003). Moreover, from an infectious disease control standpoint, grouping morphologically similar species into a single complex category function as an optimized “triage strategy.” In low-resource settings where screening budgets are heavily constrained, utilizing our algorithm as a primary screening filter to flag suspect *Entamoeba* complexes can dramatically reduce the volume, and thus the economic burden of subsequent mandatory PCR testing, allowing public health resources to be allocated more efficiently.

Furthermore, the current algorithm does not address the complete spectrum of *Blastocystis* sp. morphological forms. As *Blastocystis* sp. exists in vacuolar, granular, amoeboid, and cyst forms in stool specimens (Stensvold and Clark, 2016; Tan, 2008), the present dataset primarily reflects the vacuolar form, which represents the most epidemiologically relevant and frequently detected morphotype in routine public health screenings. While expanding the algorithm's capability to other forms is necessary for comprehensive diagnostic coverage, its current high performance on the vacuolar form ensures its immediate utility for standard field surveillance. The algorithm's performance on other morphological forms of *Blastocystis* sp. has not been evaluated and represents an area for future investigation. Additionally, a current limitation of the algorithm is its classification of *Blastocystis* sp. under noisy imaging conditions. In the proposed framework, *Blastocystis* sp. is identified only under Case 1c (Fig. 5), when solely Attribute 3 (nuclei compressed to the periphery of the cyst) is detected. However, due to image noise, Mask R-CNN may simultaneously detect Attribute 3 alongside Attribute 1 or Attribute 2, routing the cyst into one of the Two-Attribute cases (Case 2). As none of the three Two-Attribute sub-cases (Case 2a: Attributes 1&2; Case 2b: Attributes 2&3; Case 2c: Attributes 1&3) produces a *Blastocystis* sp. output, such noise-induced two-attribute detections in *Blastocystis* sp. images are not correctly resolved by the current algorithm. Specifically, Case 2b (Attributes 2&3) would incorrectly classify the cyst as *E. histolytica* or *E. hartmanni*, and Case 2c (Attributes 1&3) would incorrectly classify it as *E. nana* or *I. bütschlii*. Addressing this limitation by refining the Two-Attribute decision rules to incorporate a *Blastocystis* sp. output pathway is identified as a direction for future work.

Finally, the dataset used in this study exhibits severe class imbalance, particularly for *I. bütschlii* (*n* = 24), resulting in a limited recall of 18.2% for this class, despite achieving an exceptional precision of 100%. This stark contrast highlights the highly conservative nature of the proposed framework under data scarcity; because the expert heuristic rules enforce rigid morphological constraints to prevent false positives, the system only triggers a positive identification when a cyst perfectly satisfies all diagnostic criteria, thereby increasing the false-negative rate for underrepresented species. Future work should prioritize dataset augmentation for rare species, explore synthetic data generation strategies, and refine the heuristic routing constraints by incorporating additional biological features, such as nuclear count and glycogen vacuole characteristics, to improve classification robustness and generalizability across all parasite classes. In addition, external validation using specimens from different laboratories, staining protocols, microscope systems, and geographic settings will be necessary to assess the generalizability of the proposed framework across diverse surveillance environments. Ultimately, refining these routing constraints and diversifying the dataset's geographical representation will facilitate the transition of this knowledge-driven AI tool from a laboratory prototype to an accessible asset for primary healthcare centers, contributing significantly to global control and elimination of neglected intestinal protozoan infections.

## 5 Conclusion

In this study, we developed an automated framework that integrates deep learning-based nucleus detection with expert morphological heuristic rules to classify seven types of intestinal protozoan cysts. Validated against an expert-labeled clinical dataset, with suspected *E. histolytica* cases rigorously confirmed via Taiwan CDC PCR as the gold standard, the hybrid pipeline achieved an overall accuracy of 84.8% and an overall F1-score of 0.735. Importantly, the exceptional processing speed of 0.04 s per image positions this framework as a highly viable engine for large-scale epidemiological mapping, enabling under-resourced field clinics to achieve standardized, high-throughput screening without being constrained by the severe shortage of senior parasitologists. Crucially, the framework balances algorithmic performance with real-world clinical utility by maintaining separate classifications for morphologically similar species, ensuring that high-risk pathogens remain distinct rather than being obscured to artificially inflate accuracy. By bridging microbiological expertise with interpretable AI architectures, this system provides an efficient, transparent, and high-throughput screening and surveillance-support tool that can mitigate laboratory burden and strengthen parasite control workflows. Future work will focus on expanding the image repository and refining heuristic constraints to further enhance diagnostic robustness in resource-constrained settings, ultimately contributing to the strengthening of active disease surveillance systems and the global containment of neglected intestinal protozoan infections.

## Ethics approval, consent to participate, and consent to publish

This study was approved by the Institutional Review Board of Kaohsiung Municipal Siaogang Hospital, Taiwan, for the anonymous use of leftover human stool specimens collected by the Laboratory of Medicine of Kaohsiung Municipal Siaogang Hospital, Taiwan (Approved No. KMUHIRB-E(II)-20240334). Due to the retrospective nature of the study and the complete anonymization of the leftover clinical specimens, the institutional review board waived the requirement for individual informed consent.

## CRediT authorship contribution statement

**Je-Chiang Tsai:** Writing – review & editing, Writing – original draft, Supervision, Software, Resources, Project administration, Methodology, Investigation, Funding acquisition, Formal analysis, Conceptualization. **Yi-Ru Chen:** Software, Formal analysis. **Shu-Min Tan:** Writing – review & editing, Visualization, Software, Conceptualization. **Kai-Lun Jin:** Software. **Chih-Chi Wu:** Writing – original draft, Visualization, Formal analysis. **Jie-Hong Lai:** Software. **Kuan-Lin Huang:** Software. **Chia-Chieh Chu:** Conceptualization. **Shih-Hsun Hung:** Formal analysis. **Jiunn-Yuan Lin:** Formal analysis. **Sukhdeep Singh Bal:** Writing – review & editing. **Fan-Pei Yang:** Conceptualization. **Kang-Shuo Lee:** Data curation. **Tsai-Wei Chen:** Data curation. **Chih-Hsing Hung:** Conceptualization. **Ching-Chao Liang:** Project administration. **Huei-Jiun Tsai:** Data curation. **I-Pin Chen:** Data curation. **Chiu-Hui Li:** Data curation. **Ya-Nien Hsieh:** Data curation. **Chin-Ling Tsai:** Data curation.

## Ethics declaration

The authors declare that all methods and protocols involving leftover human specimens in this study were conducted in strict accordance with the ethical standards of the institutional research committee and the 1964 Declaration of Helsinki and its later amendments.

## Funding

This work was supported by Kaohsiung Municipal Siaogang Hospital, Taiwan, under Grant No. 114A2003I2.

## Declaration of competing interest

The authors declare that they have no known competing financial interests or personal relationships that could have appeared to influence the work reported in this paper.
